# Smaller Fixation Target Size Is Associated with More Stable Fixation and Less Variance in Threshold Sensitivity

**DOI:** 10.1371/journal.pone.0165046

**Published:** 2016-11-09

**Authors:** Kazunori Hirasawa, Kana Okano, Risako Koshiji, Wakana Funaki, Nobuyuki Shoji

**Affiliations:** 1 Department of Orthoptics and Visual Science, School of Allied Health Sciences, Kitasato University, Kanagawa, Japan; 2 Department of Ophthalmology, School of Medicine, Kitasato University, Kanagawa, Japan; Xiamen University, CHINA

## Abstract

The aims of this randomized observational case control study were to quantify fixation behavior during standard automated perimetry (SAP) with different fixation targets and to evaluate the relationship between fixation behavior and threshold variability at each test point in healthy young participants experienced with perimetry. SAP was performed on the right eyes of 29 participants using the Octopus 900 perimeter, program 32, dynamic strategy. The fixation targets of Point, Cross, and Ring were used for SAP. Fixation behavior was recorded using a wearable eye-tracking glass. All participants underwent SAP twice with each fixation target in a random fashion. Fixation behavior was quantified by calculating the bivariate contour ellipse area (BCEA) and the frequency of deviation from the fixation target. The BCEAs (deg^2^) of Point, Cross, and Ring targets were 1.11, 1.46, and 2.02, respectively. In all cases, BCEA increased significantly with increasing fixation target size (p < 0.05). The logarithmic value of BCEA demonstrated the same tendency (p < 0.05). A positive correlation was identified between fixation behavior and threshold variability for the Point and Cross targets (ρ = 0.413–0.534, p < 0.05). Fixation behavior increased with increasing fixation target size. Moreover, a larger fixation behavior tended to be associated with a higher threshold variability. A small fixation target is recommended during the visual field test.

## Introduction

Fixation behavior mainly consists of voluntary and involuntary eye movements and involves two important aspects: fixation location and fixation stability. Both voluntary and involuntary fixation behavior can be assessed with perimetry measurements. Perimetry is the systematic measurement of visual field function performed during central fixation without eye movement. Specifically, fixation monitoring during the visual field measurement is very important [1,2]. Different methods have been used for fixation monitoring during perimetry. The Humphrey field analyzer (HFA) employs the gaze tracking system and the Heijl–Krakau blind spot monitoring method [3], and the Octopus perimeter employs a video monitor with a display and an automatic eye-tracking system. Although a gaze tracking system with the HFA records the fixation behavior as a waveform by using corneal reflection [3], the amount and direction are not quantified. In addition, the gaze tracking system with the HFA records fixation only when stimuli are presented. Moreover, the gaze tracking system with the HFA does not determine the mean recording fixation from start to finish. Monitoring by an examiner by using a video monitor is a subjective method, and the automatic tracing system cannot record and quantify fixation location and stability.

Many studies have used perimetry for quantitative evaluation of fixation behavior during static perimetry [4–20]. Fixation behavior during static perimetry was larger in patients with glaucoma [4,12,14], age-related macular degeneration [14–17,19,21], diabetic maculopathy [8,14], and macular holes [18]. In addition, with a decrease in the fixation target size, fixation behavior decreased in normal participants [13,21]. However, fundus perimetry is performed using the direct projection method rather than real-space and dome-shaped instruments such as the HFA or OCTOPUS 900 perimeter. In addition, most previous studies have evaluated fixation behavior within only a 10° visual field [4,12,21,22]. Hence, fixation behavior should be quantitatively evaluated in a visual field of 30°, commonly used in clinical practice, such as implemented in the 30–2 or 24–2 program of the HFA and the 32 or G program of the Octopus perimeter.

The aims of this study were to quantify the amount and frequency of fixation behavior during standard automated perimetry (SAP) within 30° among different fixation targets and to evaluate the relationship between fixation behavior and threshold variability at each test point by using wearable eye-tracking glasses in healthy young participants with prior experience undergoing perimetry.

## Methods

This study followed the tenets of the Declaration of Helsinki. Each participant provided written informed consent after the ethics committee of Kitasato University School of Allied Health Science approved the study (No. 2015–09). This study was registered in the UMIN Clinical Trials Registry (http://www.umin.ac.jp/) under the unique trial number UMIN000018393 (date of registration: 07/23/2015). This study was performed between July 2015 and December 2015. The authors confirm that all ongoing and related trials for this drug/intervention are registered.

**Fig 1:**
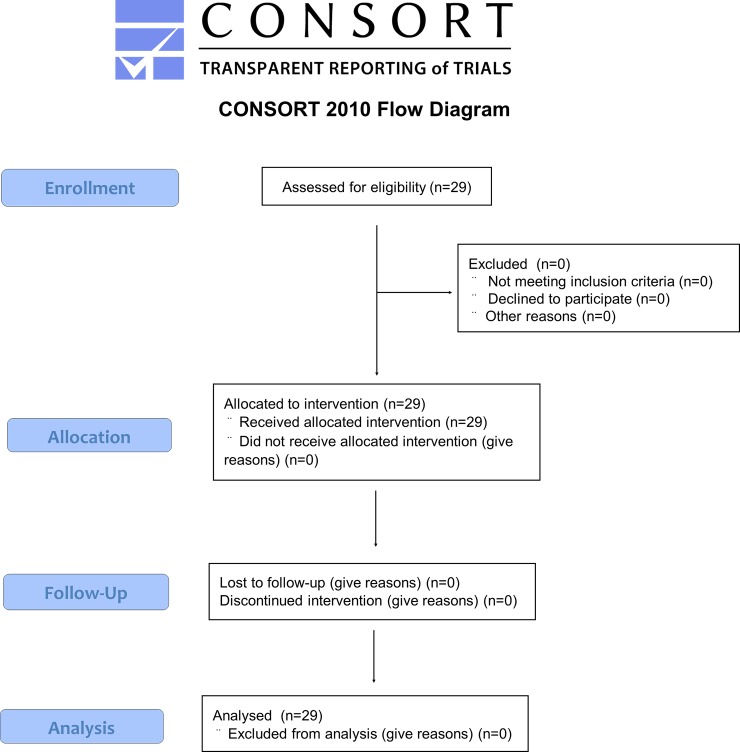
Study Protocol in this prospective observational case control study.

In this randomized observational case control study, we evaluated the right eyes of 29 student volunteers taking the Orthoptic and Visual Science course at Kitasato University who had undergone SAP at least three times within the last 3 months after providing informed consent. All the participants underwent comprehensive ophthalmic examinations, including noncycloplegic refraction testing, visual acuity testing at 5 meters using a Landolt ring chart, intraocular pressure measurement, ocular axial length measurement, and slit-lamp and fundus examination by a glaucoma specialist (NS). Participants were included in the study if they had a corrected visual acuity of 20/20 or better, intraocular pressure of 21 mmHg or less, cylindrical power of -1.50 diopter or less, a normal optic disc appearance, and no ophthalmic diseases that affected the results of the visual field test. Fig 1 shows the study protocol.

**Fig 2:**
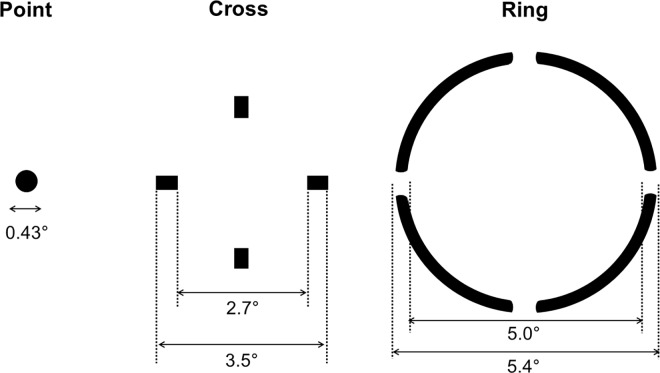
Sizes of the Point, Cross, and Ring Fixation Targets. The internal diameters and external diameters are presented for the Cross and Ring marks.

SAP was performed using the Octopus 900 perimeter (Haag-Streit, Koeniz, Switzerland). A dynamic strategy and the 32 test program were used for SAP measurement. The fixation targets of a Point mark with a visual angle of 0.43°, a Cross mark with a visual angle of 3.5°, and a Ring mark with a visual angle of 5.4° were used to obtain SAP measurements (Fig 2). The measurement condition for determining the stimulus presentation interval was set to “adaptive”. The automatic eye-tracking mode was set to “off” because reflections from the wearable eye-tracking glasses result in automatic eye tracking. The examiner corrected the pupil position manually. The maximum stimulus intensity and the stimulus presentation time were set to 10,000 asb and 0.1 sec, respectively.

**Fig 3:**
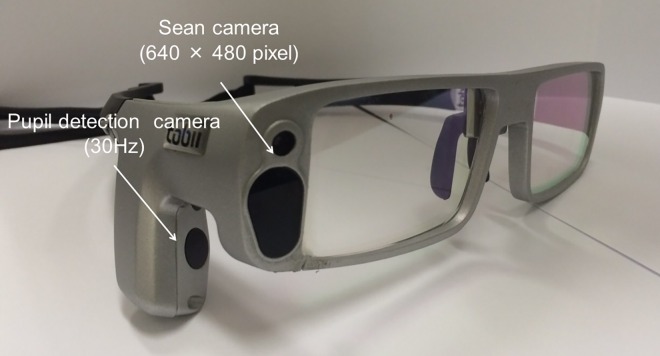
Schematic of Tobii Glass I. The wearable eye-tracking glasses consist of a camera with a resolution of 640×480 pixels mounted on the right temple, which records the eyesight of the participant; it also includes a pupil detection camera with a sampling frequency of 30 Hz located on the right temple of the glasses for recording the participant’s fixation.

Fixation behavior was recorded using wearable eye-tracking glasses (Tobii glass I; Tobii Technology). The eye-tracking glasses consist of a scene camera with a resolution of 640 × 480 pixels and a pupil detection camera with a sampling frequency of 30 Hz (Fig 3). The fixation data recorded by the two cameras were stored in the SD card of the recording assistant device. Calibration was performed with a custom 9-point grid (3 × 3 points) based on the user manual. To ensure accuracy, the calibration was performed immediately before each test and checked. Recalibration was performed if the initial calibration was inaccurate and prior to switching machines. The eye-tracking glasses record measurements for only the right eye because of the device limitation. Recorded data were exported as pixel data for the x- and y-axes. The pixel data were converted to degrees.

Measurements were obtained only for the right eye for all participants. The refractive error was corrected with disposable soft contact lens (1-Day Acuvue, Johnson & Johnson Vision Care, Inc., New Brunswick, NJ). SAP was measured using the three fixation targets marks (Point, Cross, and Ring) in a random order. Participants were allowed to rest for more than 5 minutes between sessions. After removing data corresponding to the setup and blinking, fixation behavior expressed as bivariate contour ellipse area (BCEA) and frequency of fixation behavior from the fixation target obtained during the SAP examination were analyzed for each fixation target. The logarithm of the BCEA was also calculated. The relationship between fixation behavior and threshold variability at each test point was analyzed using fixation behavior at the first measurement and the root mean square error (RMSE) value of the difference of the actual threshold at the 52nd test points excluding the extreme periphery of 30°, leaving two nasal points (two-time measurement). The following formula was used:

$$\mathrm{RMSE}=\sqrt{\sum_{i=1}^{52}{\left({Ti}_{f}-{Ti}_{s}\right)}^{2}}$$

where *Ti*_*f*_ and *Ti*_*s*_ are the threshold values of the first and second measurements of test point *i*, respectively.

BCEA was calculated for a given proportion of all fixation points using the following formula [23]:

$$\mathrm{BCEA}=2k*\sigma_{H}*\sigma_{V}*\sqrt{\left(1-\rho^{2}\right)}$$ (1)

where *σ*_*H*_ is the standard deviation of the fixation location over the horizontal meridian, *σ*_*V*_ is the standard deviation of the fixation location over the vertical meridian, and *ρ* is the product-moment correlation of these two position components. The value of k depended upon the area chosen:

$$p=1-e^{-k}$$ (2)

where *e* is the base of the natural logarithm. Therefore, 63.22% of the fixation positions lie within this area when k is 1. For the current study, fixation data were calculated with p-values of 0.6827 (k = 1.147), 0.9545 (k = 3.079), and 0.9973 (k = 5.521) corresponding to one, two, and three standard deviations of the fixation location data.

### Statistical analysis

All data were analyzed using MedCalc, version 13.2.0.0 (MedCalc Software, Ostend, Belgium), R statistical software (The R Project for Statistical Computing), and G*Power3, version 3.1.7 (Franz Faul, Universität Kiel, Germany).

The paired *t*-test or Wilcoxon signed-rank test was used for comparison of mean values between two samples. Spearman's rank correlation coefficient (ρ) was used for data correlation. The Bonferroni tests were used for data comparisons at each time point. Bonferroni-corrected probability values of < 0.05 were considered to indicate statistically significant differences. The effect size, α error, power (1−β error), and nonsphericity correction were 0.25 (middle), 0.05, 0.80, and 0.50, respectively, and the required sample size was 29 participants for 3 repeated measurements.

## Results

Table 1 shows the demographic characteristics of the participants included in the current study.

**Table 1:** Participant Demographic and Ocular Characteristics.

Parameter	Mean ± SD	Range
**Age (years)**	21.4 ± 1.1	20 to 25
**Visual acuity (LogMAR)**	-0.28 ± 0.04	-0.30 to -0.18
**Spherical equivalent (diopter)**	-2.80 ± 3.20	-10.50 to 3.63
**Cylindrical power (diopter)**	-0.24 ± 0.40	-1.50 to 0.00
**Axial length (mm)**	24.64 ± 1.51	21.41 to 27.51
**Intraocular pressure (mmHg)**	13.0 ± 2.6	10.0 to 18.3

LogMAR, logarithmic minimum angle of resolution.

Although the test duration with the Point target in the second measurement (321.9 sec) was significantly lower than that in the first measurement (335.5 sec) (paired *t*-test, p = 0.046), there was no significant difference in any other parameter between the first and second measurements.

Table 2 shows fixation behavior and its frequency for the Point, Cross, and Ring targets. The BCEA of the Point target (1.11 deg^2^) was significantly lower than that of the Cross (1.46 deg^2^) and Ring (2.02 deg^2^) targets (p = 0.025 and p = 0.006, respectively), but there was no difference between the Cross and Ring marks (p = 0.061). The log(BCEA) values for the Point, Cross, and Ring targets were -0.06, 0.09, and 0.20, respectively; the log(BCEA) values were significantly higher for larger fixation targets. The frequency of fixation locations with small fixation behavior tended to decrease with the increase in fixation target size, especially fixation behavior within 0.5° (p < 0.05). On the other hand, the frequency of fixation locations in middle to large fixation tended to increase with the increase in fixation target size, particularly above 1.0°. The fixation location was within 1° in approximately 86% of cases and within 2° in approximately 98% of cases.

**Table 2:** Comparison of Fixation Behavior and its Frequency among the Point, Cross, and Ring Targets.

Parameter	Point	Cross	Ring	p-value		
				Point and Cross	Point and Ring	Cross and Ring
**BCEA (deg**^**2**^**)**						
1SD (68.27%)	1.11 ± 0.84	1.46 ± 0.93	2.02 ± 1.82	0.025	0.006	0.061
2SD (95.45%)	2.98 ± 2.19	2.51 ± 3.25	5.43 ± 4.89	0.025	0.006	0.061
3SD (99.73%)	5.34 ± 3.92	7.05 ± 4.50	9.75 ± 8.77	0.025	0.006	0.061
**log(BCEA)**						
1SD (68.27%)	-0.06 ± 0.30	0.09 ± 0.26	0.20 ± 0.29	0.006	< 0.001	0.025
2SD (95.45%)	0.37 ± 0.30	0.52 ± 0.26	0.63 ± 0.29	0.006	< 0.001	0.025
3SD (99.73%)	0.62 ± 0.30	0.77 ± 0.26	0.89 ± 0.29	0.006	< 0.001	0.025
**Frequency of deviation from fixation target (%)**						
<0.5°	72.4 ± 21.9	67.2 ± 19.7	53.2 ± 20.8	0.665	< 0.001	0.005
0.5° to 1.0°	21.7 ± 14.8	26.2 ± 13.2	33.3 ± 10.9	0.432	0.003	0.074
1.0° to 1.5°	3.9 ± 7.0	5.2 ± 5.9	8.5 ± 9.0	1.000	0.106	0.070
1.5° to 2.0°	1.0 ± 1.8	1.2 ± 1.7	2.5 ± 3.8	1.000	0.135	0.065
≥2.0°	0.9 ± 0.7	1.4 ± 1.7	2.4 ± 3.8	0.262	0.216	0.273
**Mean deviation from fixation target (deg)**						
	0.41 ± 0.18	0.48 ± 0.16	0.60 ± 0.21	0.319	< 0.001	0.0558

The p-values were adjusted with the Bonferroni correction. BCEA, bivariate contour ellipse area.

**Fig 4:**
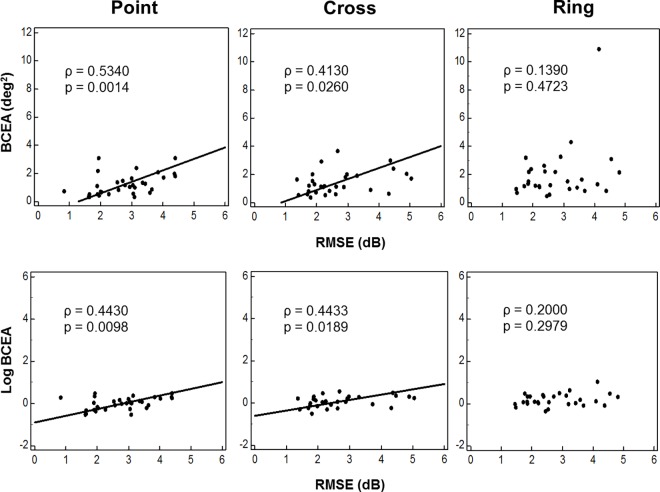
Relationship between Fixation Behavior and Threshold Sensitivity. (Top) Scatter plots of the relationship between the root mean square error (RMSE) at each test point and the bivariate contour ellipse area (BCEA). (Bottom) The relationship between RMSE and log(BCEA). Correlation coefficients (ρ) were calculated with Spearman's rank correlation coefficient.

Fig 4 shows the relationship between fixation behavior and threshold variability at each test point for the Point, Cross, and Ring targets. Fixation behavior in terms of both BCEA and log(BCEA) was significantly positively correlated with threshold variability for the Point (ρ = 0.4430 and 0.5340, p < 0.05) and Cross (ρ = 0.4130 and 0.4433, p < 0.05) targets.

**Fig 5:**
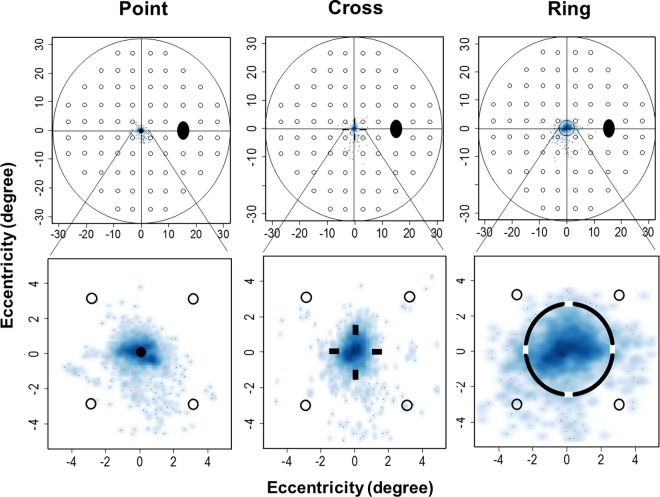
Typical Example of Fixation Behavior among the Three Fixation Targets. (Top) Fixation behavior of the visual field within 30° and (Bottom) expanded within 5°.

Fig 5 shows a typical example of fixation behavior among the three fixation targets, observed as a smooth scatter plot.

## Discussion

This randomized controlled trial study is the first to analyze the fixation behavior during SAP within 30° by using different fixation targets and to evaluate the association between fixation behavior and threshold variability by using wearable eye-tracking glasses and a real-space and domed-shape perimeter. Fixation behavior during SAP decreased with a decreasing fixation target size, and the frequency of fixation deviation from the fixation target was within 1° and 2° in approximately 86% and 98% of cases, respectively. Fixation behavior and threshold variability for the Point and Cross targets were weakly correlated.

Using fundus perimetry with Cross (4°) and Circle (4°) targets, Cesareo et al. [13] reported fixation behavior expressed as log(BCEA) to be 0.61 and 1.16, respectively, in 29 healthy participants. Although fundus perimetry performed with a built-in infrared gaze tracking camera has almost the same sampling rate as the one used in the current study (25 Hz), the fixation behavior with the Cross (log[BCEA] = 0.09) and Ring (log[BCEA] = 0.60) targets in the current study was lower than that in the study of 29 healthy participants by Cesareo et al. [13]. A possible reason for the difference could be the recording duration. Cesareo and colleagues recorded fixation behavior for 45 seconds with a sampling rate of 25 Hz, whereas in our study fixation behavior was recorded for approximately 300 seconds with a sampling rate of 30 Hz. Accordingly, the large amount of stable fixation data likely stabilized the output in the present study. In addition, the differences in methods might have influenced the results, since Cesareo and colleagues [13] used fundus perimetry, which tracks the initial reference frame of the fovea, while we used SAP, which tracks the initial reference frame of the pupil.

A study of 14 healthy participants by Lin et al. [22] reported that fixation behavior during Humphrey SAP 10–2 with Point fixation targets (approximately 1.1°) had a mean of 2.9°. Although the wearable eye-tracking glasses had the same sampling rate of 30 Hz in our study and in the study by Lin at al. [22], the fixation behavior in the current study (0.4° to 0.6°) was lower than that obtained in the study by Lin and colleagues. The difference in the results might be due to the fact that the student volunteers recruited in the current study had adequate understanding of and experience undergoing the visual field test. In addition, the visual field testing area and the shape of the wearable eye-tracking glasses were different in the two studies.

The study of large number of glaucoma patients by Ishiyama et al. [24–26] reported that the gaze tracking waveform recorded by the HFA influenced the reliability of the results of visual field tests. In particular, fixation behavior exceeding 3° tended to have a lower mean deviation value [24] and was influenced by the structure-function relationship analysis [25]. In the current study, a weak significant correlation was demonstrated between fixation behavior and threshold variability expressed as RMSE for the Point and Cross targets. A gaze tracking waveform with the HFA records fixation behavior using corneal reflection, with the forehead and chin resting on the perimeter only when stimuli are presented [3]. The fixation behavior before and after presenting the stimuli is not recorded, and it is expected that the gaze tracking waveform will record fixation behaviors other than those arising from face or forehead movement. The current results might reflect the relationship between fixation behavior and threshold variability after excluding noise because the participants wore eye-tracking glasses.

The study of 175 patients by Fujii et al. [6] yielded the following criterion for fixation reliability for MP-1: location of fixation is graded to be predominantly central (more than 50% of the points of fixation are located in the central 2°), and the stability of fixation is graded as stable (more than 75% of the points of fixation are located in the central 2°). The criteria in the study by Fujii et al. [6] were developed for age-related macular degeneration, which involves damage to the central visual field, rather than glaucoma, which does not affect the central visual field. Hence, we cannot use the Fujii criteria for glaucomatous visual field defects. Although further studies are required for glaucoma patients and normal controls, the finding that the fixation location was within 1° in approximately 86% of cases and within 2° in approximately 98% of cases for healthy young participants who had previously undergone perimetry can be used as a reference.

The study of 12 healthy participants by Thaler et al. [27] reported that the best fixation target for fixation stability was a combination of bulls-eye and cross hair targets in healthy participants. The study of 12 patients by Bellmann et al. [21] reported that the fixation behavior was significantly less often within 1° for the cross hair target compared to the four-point diamond in healthy participants. The fixation targets used in both studies are similar in the center rather than at the periphery. Although the fixation targets in the current study had different shapes, the Point target was the most stable fixation target. Hence, we recommend the use of a small fixation target, which corresponds to the center of the fixation target rather than periphery.

The test point interval used in HFA is defined by a 6° or 2° grid. In contrast, test points of the G program are distributed unevenly based on the nerve fiber layer, whereas test points of the M program are also distributed unevenly, but a 0.7° grid is used in the central area of the Octopus perimeter. In the current study, the mean deviation of the fixation location from the fixation target was approximately 0.4° to 0.6°. In other words, careful attention is required for fixation behavior because there is a potential for duplication of the stimulus area when a stimulus size of 0.43° is used in the central area in the M program of the Octopus perimeter. However, the current study did not evaluate the differences in the fixation behavior in different measurement areas, nor did it compare eye measurement data between patients with glaucoma and corresponding healthy participants. Hence, further studies are required.

## Conclusions

This is the first study to evaluate fixation behavior during real-space and dome-shape SAP within 30° using different stimulus targets; it is also the first study to evaluate the relationship between fixation behavior and threshold variability. In healthy young participants with prior experience of undergoing perimetry, fixation behavior increased with an increasing fixation target size. Moreover, a larger fixation behavior tended to be associated with a higher threshold variability, although this correlation, when present, was weak.

## Supporting Information

S1 Supporting Information Trend checklist. (PDF)

S2 Supporting Information Study protocol and approval document by institutional review board. (DOCX)
