# Supplementary material for: Smaller Fixation Target Size Is Associated with More Stable Fixation and Less Variance in Threshold Sensitivity
Source: PLoS One. 2016 Nov 9;11(11):e0165046. doi: 10.1371/journal.pone.0165046 (PMC5102375; doi:10.1371/journal.pone.0165046)
Supplement: S2 Supporting Information — (DOCX) [file pone.0165046.s002.docx]

**Study protocol**

***Study title:*** Investigation of fixation behavior during standard automated perimetry

***Applicant:*** Nobuyuki Shoji

Professor of Allied Health Sciences, Kitasato University

***Research collaborator:*** Kazunori Hirasawa, Kana Okano, Risako Koshiji, and Wakana Funaki

***Term of Study:*** Date of approval to March 31st 2019

***Type of Study:*** non-intervention and non-invasion study

***Study location:*** A3 building at Allied Health Sciences

***Protection of personal data:*** Anonymization

***Responsible person of personal data:*** Prof. Nobuyuki Shoji

***Reward for study cooperation:*** None

***Disclosure of analyzed data:*** Not to disclose all data due to anonymization

***Estimated sample size:*** Fifty eyes of 50 healthy participants

***Purpose of study:*** Fixation monitoring during the visual field measurement is very important. The Humphrey field analyzer (HFA) employs the gaze tracking system and the Heijl–Krakau blind spot monitoring method, and the Octopus perimeter employs a video monitor with a display and an automatic eye-tracking system. Although a gaze tracking system with the HFA records the fixation behavior as a waveform by using corneal reflection, the amount and direction are not quantified. In addition, the gaze tracking system with the HFA records fixation only when stimuli are presented. Hence, fixation behavior should be quantitatively evaluated in a visual field of 30°, commonly used in clinical practice, such as implemented in the 30-2 or 24-2 program of the HFA and the 32 or G program of the Octopus perimeter.

The aims of this study were to quantify the amount and frequency of fixation behavior during standard automated perimetry (SAP) within 30° among different fixation targets and to evaluate the relationship between fixation behavior and threshold variability at each test point by using wearable eye-tracking glasses in healthy young participants with prior experience undergoing perimetry.

***Methods:*** We evaluate the student volunteers taking the Orthoptic and Visual Science course at Kitasato University who had undergone SAP at least three times within the last 3 months after providing informed consent. All the participants undergo comprehensive ophthalmic examinations, including noncycloplegic refraction testing, visual acuity testing at 5 meters using a Landolt ring chart, intraocular pressure measurement, ocular axial length measurement, and slit-lamp and fundus examination by a glaucoma specialist (NS). Participants are included in the study if they had a corrected visual acuity of 20/20 or better, intraocular pressure of 21 mmHg or less, cylindrical power of -1.50 diopter or less, a normal optic disc appearance, and no ophthalmic diseases that affected the results of the visual field test.

SAP is performed using the Octopus 900 perimeter (Haag-Streit, Koeniz, Switzerland). A dynamic strategy and the 32 test program were used for SAP measurement. Fixation behavior is recorded using wearable eye-tracking glasses (Tobii glass I; Tobii Technology).

***Submission date:*** May 11th, 2015

***Acceptance date:*** July 1st, 2015

This study was approved by Kitasato University School of Allied Health Science approved the study (No. 2015-09)
